# Genome-Wide Association Study Reveals *PC4* as the Candidate Gene for Thermal Tolerance in Bay Scallop (*Argopecten irradians irradians*)

**DOI:** 10.3389/fgene.2021.650045

**Published:** 2021-07-19

**Authors:** Xinghai Zhu, Pingping Liu, Xiujiang Hou, Junhao Zhang, Jia Lv, Wei Lu, Qifan Zeng, Xiaoting Huang, Qiang Xing, Zhenmin Bao

**Affiliations:** ^1^MOE Key Laboratory of Marine Genetics and Breeding, College of Marine Life Sciences, Ocean University of China, Qingdao, China; ^2^Laboratory for Marine Fisheries Science and Food Production Processes, Qingdao National Laboratory for Marine Science and Technology, Qingdao, China

**Keywords:** genome-wide association study, *Argopecten irradians irradians*, thermal tolerance, single nucleotide polymorphism, *transcriptional coactivator p15*

## Abstract

The increasing sea temperature caused by global warming has resulted in severe mortalities in maricultural scallops. Therefore, improving thermal tolerance has become an active research area in the scallop farming industry. Bay scallop (*Argopecten irradians irradians*) was introduced into China in 1982 and has developed into a vast aquaculture industry in northern China. To date, genetic studies on thermal tolerance in bay scallops are limited, and no systematic screening of thermal tolerance-related loci or genes has been conducted in this species. In the present study, we conducted a genome-wide association study (GWAS) for thermal tolerance using the Arrhenius break temperature (ABT) indicators of 435 bay scallops and 38,011 single nucleotide polymorphism (SNP) markers. The GWAS identified 1,906 significant thermal tolerance-associated SNPs located in 16 chromosomes of bay scallop. Gene ontology and Kyoto Encyclopedia of Genes and Genomes pathway analyses showed that 638 genes were enriched in 42 GO terms, while 549 annotated genes were enriched in aggregation pathways. Additionally, the SNP (15-5091-20379557-1) with the lowest *P* value was located in the *transcriptional coactivator p15* (*PC4*) gene, which is involved in regulating DNA damage repair and stabilizing genome functions. Further analysis in another population identified two new thermal tolerance-associated SNPs in the first coding sequence of *PC4* in bay scallops (*AiPC4*). Moreover, *AiPC4* expression levels were significantly correlated (*r* = 0.675–0.962; *P* < 0.05) with the ABT values of the examined bay scallops. Our data suggest that *AiPC4* might be a positive regulator of thermal tolerance and a potential candidate gene for molecular breeding in bay scallop aiming at thermal tolerance improvement.

## Introduction

Aquatic products constitute an important source of food consumed all over the world. As of 2019, global aquaculture output was 80.1 million tons (46.8 million tons contributed by China), generating an estimated 238.0 billion dollars (139.1 billion dollars by China) in revenue (FAO, 2019). Many bivalves are economically important aquaculture species that account for approximately 22.8% of the aquatic products market worldwide (Moffitt and Cajas-Cano, 2014). Although a continuous increase in the production of bivalves has been observed in recent years (Steeves et al., 2018), several obstacles (e.g., environmental stress, biotic and abiotic factors) hamper the development of the industry. Many studies revealed that, anoxia tolerance (Zwaan et al., 1995; Chen et al., 2007a), salinity tolerance (Chen and Chen, 2000; Roldan-Carrillo et al., 2005), and tolerance to bacterial pathogens (Richards et al., 2015; Rojas et al., 2016) are closely related to mortality in bivalves, which may have adverse effect on global aquaculture production. Consequently, researchers gave more attention on the tolerance ability of aquatic commercial bivalves for sustainable development of shellfish industry.

Bivalves are poikilotherms, therefore, temperature is one of the most important environmental factors affecting their physiology (Zittier et al., 2015), including growth efficiency (Sicard et al., 1999), reproductive activity (Martínez and Pérez, 2003), immune defense (Chen et al., 2007b), tolerance ability (Chen and Chen, 2000), and even leading to death (Cheng et al., 2020) in bivalves. For instance, the daily shell growth rate of Antarctic scallops (*Adamussium colbecki*) during summer was severely affected by temperature (Heilmayer et al., 2005). During the reproductive cycle of mussel (*Mytilus edulis*), variations in temperature significantly affected the development of the gonads (Lubet, 1983). An increase in temperature significantly decreased the activities of its hemocytes, and consequently increased the mortality rate of Pacific oysters (Gagnaire et al., 2006). Most importantly, thermal tolerance in scallops has been a major target trait in breeding because massive mortality usually occurs in summer. High temperature of seawater in summer even caused a 37% decline in the production of Zhikong scallop (MAC, 1999). Consequently, accurate and efficient indicators for thermal tolerance evaluation have been playing a crucial role in accelerating the breeding progress of thermal resistant scallops (Mason, 1958; Granados-Amores et al., 2012; Yang et al., 2013; Xing et al., 2016a; Ning et al., 2019). Specifically, the heart rate (HR)-based Arrhenius break temperature (ABT), the temperature at which the Arrhenius plot exhibits a sharp discontinuity in slope, is regarded as a heterothermic organism’s upper limit temperature (Dahlhoff et al., 1991) and could represent species’ maximal habitat temperature (Weinstein and Somero, 1998). ABT was originally reported in porcelain crabs (Bamber and Depledge, 1997), followed by intertidal animals (Somero, 2002), snails (Stenseng et al., 2005), and fish (Casselman et al., 2012). Xing et al. (2016a) first reported the feasibility of ABT as a rapid, efficient, and non-invasive indicator for bivalve thermal tolerance evaluation (Xing et al., 2016a). Specifically, ABT could accurately indicate thermal limits in interspecific scallops with different thermal adaptation, with different ABT values of 22.03 ± 0.19, 29.10 ± 0.25, 32.20 ± 0.25 and 34.09 ± 0.19°C for *Patinopecten yessoensis*, C*hlamys farreri*, *Argopecten irradians*, and *A*. *ventricosus*, respectively. In intraspecific Zhikong scallops, ABT negatively correlated with sizes, weights and ages of individuals, apparently differed between sexual adults, and was significantly affected by spawning behavior and seasonal variations of seawater temperature (Xing et al., 2016a, 2021). As a consequence, ABT has been used as an efficient indicator in predicting the thermal performance of shellfish (Chen et al., 2016; Xing et al., 2019, 2021), and can also be used as an indicator in the breeding of new variants of thermal resistant aquatic species.

Owing to the fast growth rate and high production of the bay scallop (*A. irradians irradians*), it has become one of the most commercially important shellfish in China since its introduction from the US in the early 1980s (Wang et al., 2007). However, in the past decades, the scallop production industry has suffered high summer mortalities, and the high temperature has been suspected to be one of the main environmental inducers (Xiao et al., 2005; Jonasson et al., 2007). As reported, compared with the heat-resistant scallop *A. irradians concentricus* distributed in southern China, scallop *A. irradians irradians* distributed in northern China exhibited a lower thermal tolerance (Liu et al., 2020). Using the shell height as the target trait, genome-wide genotyping method 2b-RAD (Wang et al., 2012), RADtyping software (Fu et al., 2013) and genomic selection (GS) strategies (Sonesson and Meuwissen, 2009; Wang et al., 2018b), researchers have been able to develop the new bay scallop strain named “Haiyifeng 11.” Interestingly, the non-invasive thermal tolerance indicator ABT (Xing et al., 2016a), regarding as thermal limit of the new strain was 34.54 ± 0.53°C, which is approximately 2°C higher than that of the common population. Accordingly, the survival rate of the thermal tolerant strain (81.32%) increased by 13.22% compared to the control group (68.10%). The thermal tolerance of “Haiyifeng 11” improved markedly after four consecutive generations of breeding *via* GS. However, the major loci or genes and molecular mechanisms underlying thermal tolerance in bay scallop are yet to be identified. In practical application, knowledge of the loci or genes regulating thermal tolerance could be used as potential markers in target genotyping technology, e.g., high-throughput, sequencing-based GoldenGate approach (HD-marker) for scallop selection breeding with better thermal tolerance performance (Lv et al., 2018).

With the advancement in technology, genome-wide association studies (GWAS) have been successfully utilized in identifying loci/genes involved in target polygenic traits; GWAS is a valuable tool for genetic improvement of target phenotypic traits (Korte and Farlow, 2013). In aquatic species, GWAS has been widely used in investigating economically phenotypic traits, especially in economic fishes, including head size (Geng et al., 2016), disease resistance (Zhou et al., 2017), heat endurance (Jin et al., 2017) and hypoxia tolerance (Wang et al., 2017). Besides, GWAS revealed that two SNPs positioned in *Salmo salar* chromosome 26 showed strong associations to its flesh color (Helgeland et al., 2019), and five SNPs distributed in four chromosomes were strongly associated with acute heat tolerance trait in large yellow croaker (Wu et al., 2021). Except for fish, GWAS has also been proven to be an efficient approach to reveal trait-associated genes and/or markers in shellfish. In Zhikong scallop (*C. farreri*), GWAS identified two growth-related quantitative trait loci and one potential sex-determination region (Jiao et al., 2014). In Yesso scallop (*P. yessoensis*), GWAS identified *PyBCO-like 1* (beta-carotene oxygenase like 1) associated with carotenoid coloration (Li et al., 2019a), *E2F3* (E2F transcription factor 3) which may regulate its growth (Ning et al., 2019), and three candidate genes involved in carotenoid metabolism, which are necessary for the formation of reddish-orange shells (Zhao et al., 2017). Additionally, GWAS analysis of heat tolerance in Pacific abalone revealed that 27 SNPs significantly affected its adhesion ability (Yu et al., 2021). These results provide a list of candidate loci/genes for functional annotation, decipher the genetic architecture of complex traits and help in identifying genetic variants that can be directly used for genetic improvement.

In the present study, we aimed to identify significant SNP markers associated with thermal tolerance in bay scallops by performing GWAS, with the aid of the non-invasive thermal tolerance indicator ABT. GWAS showed that the highest potential locus was located in the gene encoding transcriptional coactivator p15 (PC4), which repairs DNA damages and stabilizes genome function (Das et al., 2006). Gene ontology (GO) and Kyoto Encyclopedia of Genes and Genomes (KEGG) pathway analyses were further carried out. Subsequent sequencing analysis was conducted in another population to verify the SNPs. We further investigated the expression patterns of the *PC4* gene (*AiPC4*) in the tissues of bay scallop and detected a correlation between the expression levels of *AiPC4* and ABT values in bay scallops. Our results revealed the importance of *AiPC4* in thermal tolerance in bay scallops, and provided valuable loci and candidate genes for the breeding of thermal tolerant scallops.

## Materials and Methods

### Scallop Management and Miso-RAD Library Construction

“Haiyifeng 11” new strain was artificially bred from 2011 to 2016 by our group and was used for GWAS in the present study. The detailed procedure of artificial selection for “Haiyifeng 11” could be obtained in Supplementary File 1. Briefly, the new strain was initially bred from the founder population of 1,000 bay scallops, which consisted of 500 black-brown shell color individuals from Laizhou, Yantai (37°10′57 N, 119°56′55 E, Shandong Province, China) and another 500 purple-red shell color individuals from Jiaonan, Qingdao (35°52′20 N, 120°2′47 E, Shandong Province, China) in 2011. Subsequently, the new strain was bred *via* four consecutive generations by GS focusing on traits of shell height from 2012 to 2016.

Healthy 10-month-old “Haiyifeng11” bay scallops (*N* > 1,000) were contemporaneously breeding populations and were collected from artificial scallop-rearing substrates at Qingdao Jinshatan Fishery Group Co., Qingdao (35°57′23 N, 120°27′28 E, Shandong Province, China) in 2016. After sample collection, encrusted organisms on scallop shells were removed and the scallops were brought to the laboratory following standard procedures (Maeda-Martínez et al., 2000). The scallops were placed in plastic tanks containing filtered and aerated seawater (consistent with natural seawater temperature) for 7 days for acclimation (Bakhmet, 2005). To avoid tank effects, all collected scallops were randomly maintained in several plastic tanks and the water was partially replaced (1/2) daily. The animals were maintained without feed to avoid specific dynamic actions (Secor, 2009). Subsequently, we randomly selected 435 scallops and determined their ABT values using the method described by Xing et al. (2016a). Briefly, the heart rate (HR) of 435 individuals were measured to calculate ABT values. To detect the HR, the non-invasive method was employed, in which CNY-70 optical sensors were fixed with glue (Krazy glue, Westerville, United States) to the shell surface close to cardiocoelom of the scallops. Variations in the infrared signal were amplified, filtered and recorded using AMP03 (Newshift, Lisbon, Portugal) and Powerlab (8/35, ADInstruments, Sydney, Australia). Data were viewed and analyzed using LabChart software (ADInstruments, Sydney, Australia). ABT was calculated using regression analyses to generate the best fit lines on both sides of a putative break point (Dahlhoff et al., 1991; Stillman and Somero, 1996). Then, gills were removed from each scallop following the procedure described by Mao et al. (2013) and then preserved in 100% ethanol at −20°C for genomic analysis. Our experiments were conducted according to the guidelines and regulations established by the Ocean University of China and the local government.

Genomic DNA was extracted from the gills using a standard phenol-trichloromethane method with some modification (Sambrook et al., 1989). Subsequently, libraries were constructed using the Multi-isoRAD method developed by Wang et al. (2016). Briefly, genomic DNA of the 435 selected bay scallops was digested with *Bsa*XI (New England BioLabs, cat. no. R0609) at 37°C for 45 min. Adaptor ligation, PCR amplification, digestion, and ligation were carried out, after which five DNA fragments were set as one group (barcoding and pooling). After amplifying twice, each production of five concatenated tags from five samples was purified using the MinElute PCR Purification Kit (Qiagen, cat. no. 28004) and pooled for pair-end sequencing on the Illumina HiSeq2000 platform.

### Sequence Data Processing and Genotyping

To obtain high-quality reads, the raw reads were first preprocessed to remove paired-end (PE) reads with ambiguous base calls (N), long homopolymer regions (>10 bp), and low-quality bases (> 20% of bases with quality score < 10). After matching the forward and reverse reads, PEAR software was used to assemble the PE reads (Zhang et al., 2014). The reads containing five tags, which were chosen by the assembly step, were divided into single-tag datasets. The single tag containing the restriction enzyme site, extracted from single-tag data, was aligned to the chromosome-level reference genome constructed by our group (unpublished) using SOAP (Li et al., 2009). Genotyping analysis of sequencing data was performed using the RADtyping software developed by Fu et al. (2013). To generate genotypes sets for GWAS, only SNPs without missing values (SNPs with coverage < 4 were considered missing), call rate > 60%, and minor allele frequency (MAF) > 0.01 were used for downstream analysis.

### Genome-Wide Association Study and Identification of Candidate Genes

Association analysis between genotypes and ABT values was conducted using Plink-1.0.7 software (Purcell et al., 2007) by performing a Wald test using a general linear model (GLM). The Wald statistic *P*-value for each SNP was summarized in ascending order. The sequence tag of SNPs with *P* < 0.001 was extracted for BLASTN (Altschul et al., 1997) search against the draft genome of *A. irradians irradians* (unpublished data) that were annotated by the protein database NR using the code: blastall −p blastn −i input −d genome −o result −a 5 −e 1e-4 −m 8. Additionally, Manhattan plot was generated using “qqman” function on R package (version 3.6.3). Potential SNPs with *P* < 0.05 were captured for bioinformatics analysis, including GO function annotation and KEGG pathway enrichment analyses^1^.

### SNP Verification

To verify the association between the ABT values and the gene harboring the most significant SNP, another separate cultured population established by artificial fertilization with over 1,000 sexually mature bay scallops at Marine Seed Co., Ltd., Yenta (37°18′18 N, 119°52′29 E, Shandong Province, China) in 2018 was used for screening thermal tolerance associated mutations in *AiPC4*. From 150 randomly collected individuals in this population, the top 15 (10%) and the bottom 15 (10%) scallops were selected based on their ABT values in decreasing order. Then, the genomic DNA of the 30 scallops was extracted, and the three-prime UTR (chr15-5091-20379557-1), coding sequence (CDS) 1 and 2 (*AiPC* contains two CDS) of *AiPC4* were amplified using the primer pairs listed in Table 1. The PCR reaction of the above three target DNA fragments was carried out as follows: 2 μL of template DNA (50 ng/μL), 15 μL of Q5 High-Fidelity 2 × Master Mix (New England BioLabs, cat. no. M0492S), 0.4 μL (10 pmol/μL) of each forward and reverse primer, and sterile water up to 30 μL volume was added for amplifying. The related parameters in the PCR reaction condition was as follows: pre-denaturing at 98°C for 30 s, followed by denaturing at 98°C for 10 s, annealing at 60/56/56°C for 15/15/20 s, and extension at 72°C for 10/10/20 s, respectively. After 25 cycles, the sample was extended to 72°C for 2 min. The PCR products were sequenced using the Sanger method by Sangon Biotech (Shanghai, China). Sequences amplified by the same primer pair were compared among the 30 scallops using the ClustalW2 multiple alignment program^2^. When a mutation was detected, comparisons of genotype frequencies between the top 15 and the bottom 15 scallops were performed using Fisher’s exact test to identify the thermal tolerance-associated mutation. *P* values < 0.05 were considered statistically significant.

**TABLE 1:** Sequences of primers used in this study.

SNP/Gene	Primers sequence	Note
chr15-5091-20379557-1	F: 5′-TGATGTCGTCGTTGTTGC-3′	SNP verification
	R: 5′-ACTACAACCCCGTCTCCT-3′	SNP verification
CDS 1 (*AiPC4*)	F: 5′-TGAAACTTGTCATCACGCAAT-3′	SNP verification
	R: 5′-ACACTGTTAGCCTACTTATCCGA-3′	SNP verification
CDS 2 (*AiPC4*)	F: 5′-TCTCTGGTTTTTGTGTTTTGTTTA-3′	SNP verification
	R: 5′-CGATTTAGTTTTCTGAGAGTTAGC-3′	SNP verification
*AiPC4*	F: 5′-TGAGGATGAAGAAGATGGTCAAG-3′	qRT-PCR
	R: 5′-CAGAGAAGTCCCGAATATCAACA-3′	qRT-PCR

### Candidate Gene Evaluation

To investigate the expression pattern of *AiPC4* and further analyze the correlation between expression levels of *AiPC4* and thermal tolerance in bay scallops, 18 randomly selected bay scallops from another separate population (*N* > 500) at Marine Seed Co., Ltd., Yenta (37°18′18 N, 119°52′29 E, Shandong Province, China) in 2020 were recruited and their tissues (mantle, gill, gonad, kidney, hepatopancreas, striated muscle, and heart) were sampled. Total RNA of the sampled tissues was isolated following the method described by Hu et al. (2006) and then digested with DNase I (TaKaRa, Shiga, Japan). The concentration and purity of the RNA were determined using a Nanovue Plus spectrophotometer (GE Healthcare, NJ, United States), and RNA integrity was assessed by agarose gel electrophoresis. First-strand cDNA was synthesized according to the manufacturer’s protocol using Moloney murine leukemia virus (MMLV) reverse transcriptase (Thermo, United States) in a 20 μL volume with 2 μg of each total RNA sample as the template and 0.5 μg of oligo (dT)18 (TaKaRa Biotechnology, Liaoning, China) as the primer. The mixture was denatured at 65°C for 5 min and then chilled immediately on ice. After adding the reverse transcriptase, reaction buffer, and dNTPs, cDNA was amplified under the following conditions: 42°C for 90 min and 72°C for 10 min. The cDNA was stored at −20°C and diluted to 5 ng/μL for use as the template in quantitative reverse-transcription PCR (qRT-PCR).

The expression levels of *AiPC4* in the tissues of the 18 bay scallops were analyzed by qRT-PCR. The first strand cDNA from the tissues was used as the template and specific primers were designed to amplify the *AiPC4* fragments. Elongation factor 1 alpha (EF1α) was selected as a reference for tissue samples (Li et al., 2019b). The primers used for the qRT-PCR are listed in Table 1. Three technical repeats were performed for each reaction. All qRT-PCR reactions were performed using a LightCycler 480 system (Roche Diagnostics, Mannheim, Germany) with a total volume of 20 μL containing 1 × Real-time PCR Master Mix containing SYBR Green dye (TOYOBO, Osaka, Japan), 0.2 μM of each primer, and 2 μL cDNA template. The qRT-PCR reaction was carried out as follows: denaturation at 50°C for 2 min, 94°C for 10 min, followed by 40 cycles of 94°C for 15 s and 62°C for 1 min. Dissociation (from 94°C to 62°C) analysis of the amplification products was performed at the end of each PCR reaction to confirm that only one PCR product was amplified and detected.

Data from the real-time PCR were obtained, and the gene *EF1A* (elongation factor 1α) was used as an internal control. Fold-change of *AiPC4* expression levels among tissues was conducted with the gene expression level in gill as the reference. Statistical analysis of the data was performed with SPSS software 21.0 (IBM Co., Armonk, United States) *via* one-way ANOVA followed by a *post hoc* test. We used bivariate correlation analysis to determine the relationships between *AiPC4* expression levels in scallop tissues and the ABT values. Differences were considered significant if *P* < 0.05.

## Results

### ABT Values and Sequencing Data

The ABT values of the 435 bay scallops varied from 33.69 to 35.73°C, with a mean value of 34.65 ± 0.40°C. The ABT values basically follow the law of normal distribution, which is necessary for the subsequent GWAS analysis for quantitative traits (Supplementary Figure 1).

In total, 350.54 Gigabyte (G) clean data of 87 Multi-isoRAD libraries generated 1,949,337,356 reads after sequencing (GenBank: PRJNA689862). The average sequencing depth was 17.2×. After quality control, we obtained 1,908,407,252 high-quality reads, and an average of 45.15% high-quality reads of each individual were uniquely mapped to the bay scallop genome (unpublished data) by SOAP. We genotyped 489,750 SNPs in 435 individuals using RADtyping. Specifically, population genetic analysis *via* PCA analysis showed that the 435 individuals have no population stratification which is critical for subsequent GWAS.

### Genomic Regions Associated With ABT

**FIGURE 1:**
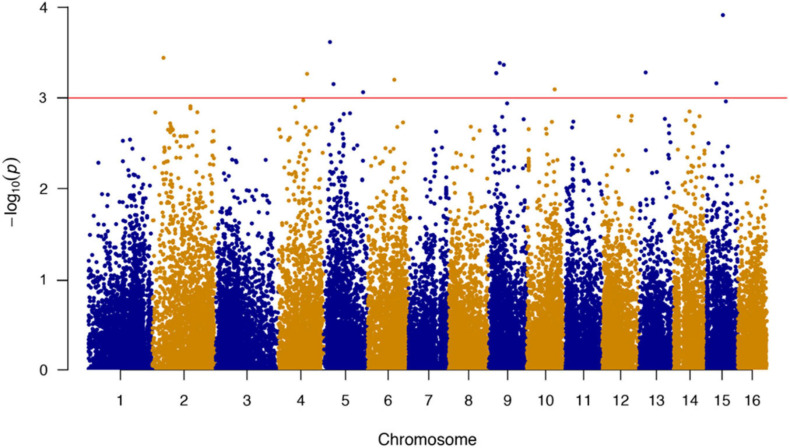
Genome-wide association study (GWAS) of thermal tolerance traits with 38,079 SNPs in bay scallop. The red line indicates the genome-wide threshold as *P*-value of 0.001.

A total of 489,750 SNP markers obtained from 435 bay scallops were subjected to quality control using PLINK software. After screening for call rate (> 60%) and MAF (> 1%), a set of 38,079 high-quality SNPs were used for GWAS. We successfully mapped 38,011 of these SNPs in the 16 chromosomes of bay scallop. The results of the GWAS showed that 1,906 SNPs were significantly (*P* < 0.05) correlated with the ABT and 1,165 of these SNPs in the 27 bp tags were directly annotated in bay scallop genome. The top 14 significant (*P* < 0.001) SNPs are shown in Table 2. Among the top 14 SNPs, the most significantly associated SNP (chr15-5091-20379557-1), which was located on chromosome 15 (Figure 1), was selected for verification by sequencing the PCR product using Sanger method (Sangon Biotech, Shanghai, China).

**TABLE 2:** Information of the top fourteen SNPs associated with ABT.

SNP	Chromosome	Allele	*P* value	Position (bp)
chr15-5091-20379557-1	15	A	1.22E-04	20379558
chr5-1950-7995894-22	5	T	2.42E-04	7995916
chr2-6408-13767598-12	2	C	3.61E-04	13767610
chr9-3029-13982597-15	9	T	4.12E-04	13982612
chr9-4119-18978396-13	9	C	4.32E-04	18978409
chr13-2659-8209392-15	13	C	5.24E-04	82093407
chr9-2238-9737120-24	9	A	5.33E-04	9737144
chr9-2238-9737120-26	9	G	5.33E-04	9737146
chr4-8816-35428563-21	4	C	5.43E-04	35428584
chr6-9827-33018625-15	6	G	6.30E-04	33018640
chr15-3384-12393137-1	15	A	6.90E-04	12393138
chr5-2940-12342236-22	5	T	7.04E-04	12342258
chr10-9342-34462988-19	10	T	8.05E-04	34463007
chr5-12284-48674468-3	5	C	8.65E-04	48674471

### SNP Verification

**FIGURE 2:**
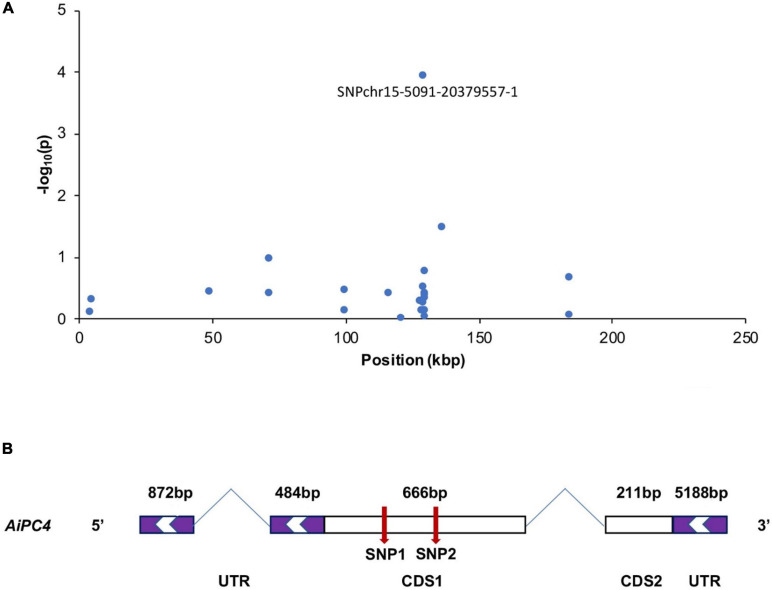
A few neighboring SNP loci on chromosome 15 are independently associated with ABT in bay scallop **(A)**. The genic structure of *AiPC4* **(B)**. The purple box indicate the 5′UTRs and 3′UTRs (untranslated region); the white boxes represent CDS (coding sequence); and the red arrows indicate the two verified SNPs. The CDS are shown relative to their lengths and the two indicated SNPs are marked in the accurate positions in the genic sequences.

The most significantly associated SNP, chr15-5091-20379557-1, was assigned to a gene encoding PC4, which is involved in repairing DNA damages and stabilizing genome function (Das et al., 2006), which may play an important role in thermal tolerance. And this SNP on *AiPC4* gene explained 5.41% variance of the total phenotypic variation according to the GWAS result. To determine the relationship between *AiPC4* and thermal tolerance in scallops, mutations spanning the transcribed sequence of *AiPC4* were screened in the top 15 (ABT = 33.19 ± 1.57°C) and bottom 15 (ABT = 30.47 ± 1.75°C) individuals (based on ABT values) from 150 randomly collected scallops (ABT values ranged from 28.32 to 35.22°C, with a mean value of 31.43 ± 1.67°C). We identified two SNPs, including c.234T < C and c.369A < G in the CDS 1 of *AiPC4* (Figure 2). Comparison between the top and bottom groups showed that the genotype frequencies at the two SNPs were significantly different (*P* = 0.008 and 0.015, respectively) (Table 3). These results support the findings of GWAS that the *PC4* locus might be involved in regulating thermal tolerance in bay scallops.

**TABLE 3:** Comparison of genotype frequencies of SNPs in *AiPC4* CDS 1 between the top (*N* = 15) and the bottom (*N* = 15) scallop groups classified by the ABT values of thermal tolerance.

Gene	Location	Locus	Genotype	Number of scallop		Fisher’s exact test *P* value
				Top group	Bottom group	
*AiPC4*	CDS 1	c.234T < C	TT	2	0	0.008
			CT	12	7	
			CC	1	8	
	CDS 1	c.369A < G	AA	3	1	0.015
			AG	11	6	
			GG	1	8	

### Identification of Candidate Genes

**FIGURE 3:**
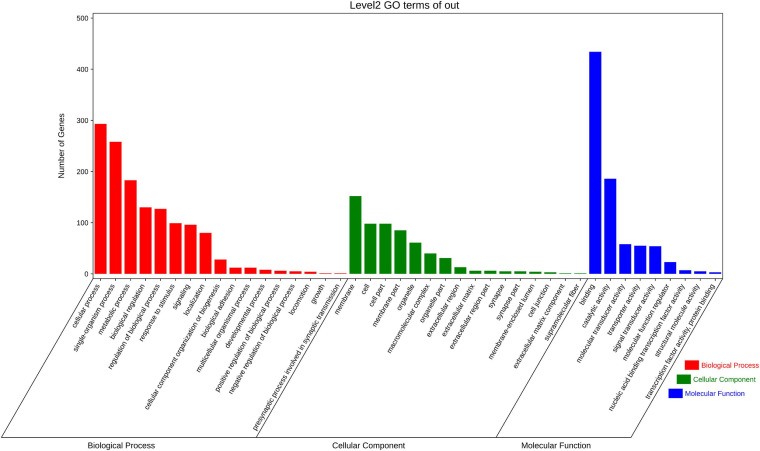
The summary of GO function annotation analysis for candidate selective genes that comprised significant SNPs (*P* < 0.05). Each term described the function of gene cluster and the length of colored bars represent the numbers difference of genes.

**FIGURE 4:**
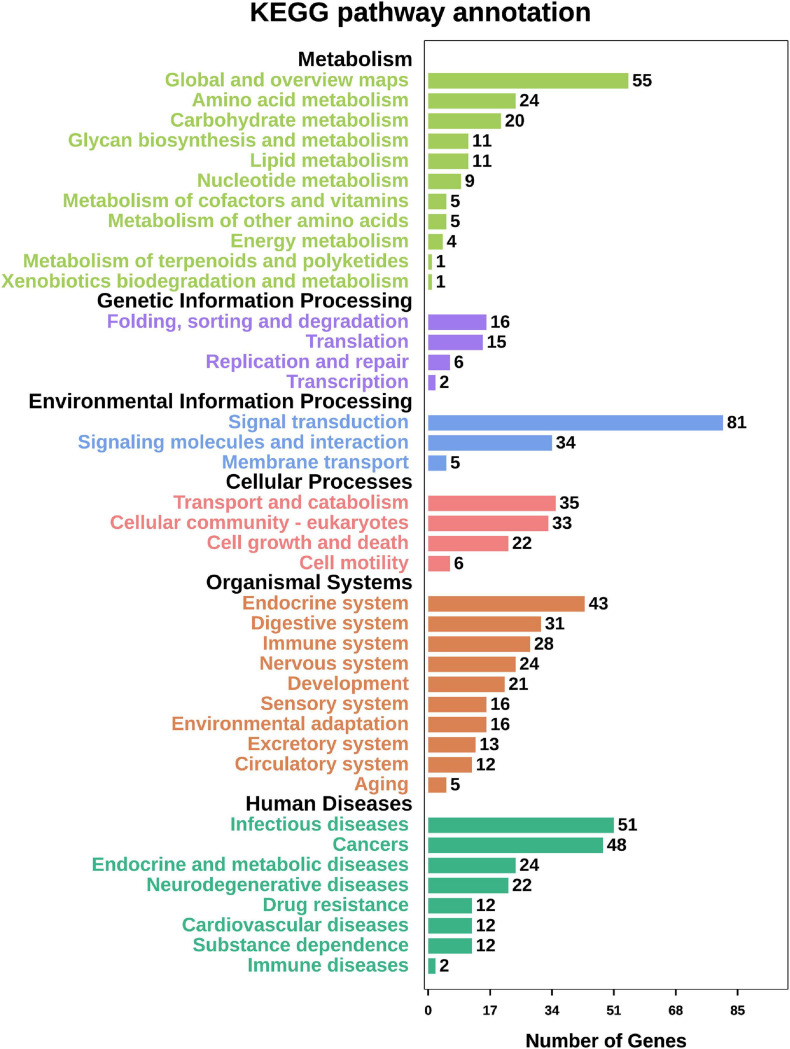
The summary of KEGG pathway enrichment analysis performed for biological process, cellular component and molecular function. Functional annotation of candidate selective genes that comprised significant SNPs (*P* < 0.05).

Among the top 14 SNPs, nine were identified in the following gene regions; *PC4*, *MYHC* (*myosin heavy chains*), *CTLM* (*ste18p G*γ *subunit*), *ALDH* (*aldehyde dehydrogenases*), *PDE11* (*phosphodiesterase 11*), *BCL7* (*B cell leukemia/lymphoma 7*), *SCAD* (*short-chain acyl-CoA dehydrogenase*), *RALDH1* (*retinaldehyde dehydrogenase family 1*), and one uncharacterized gene (*LOC110459767*). At a less stringent significance level (*P* < 0.05), there were 1,906 SNPs annotated in 950 genes. To investigate the function of these genes, we conducted GO function annotation and KEGG pathway enrichment analyses. The results showed that 638 genes were enriched in 42 GO terms (level 2) (Figure 3), and the dominant terms were cellular process (293 genes) in biological process, membrane (152 genes) in cellular component, and binding (434 genes) in molecular function. The results of KEGG pathway enrichment analysis showed that 549 genes were enriched in metabolism, genetic information processing, environmental information processing, organismal systems, and human disease (Figure 4). It should be noted that both the KEGG pathway annotation and the GO terms showed a similar trend, with environmental information processing to be referred as the most significantly enriched, including “signal transduction,” “signal molecular and interaction,” and “membrane transport.”

### *AiPC4* Expression Patterns and Its Involvement in Thermal Tolerance

**FIGURE 5:**
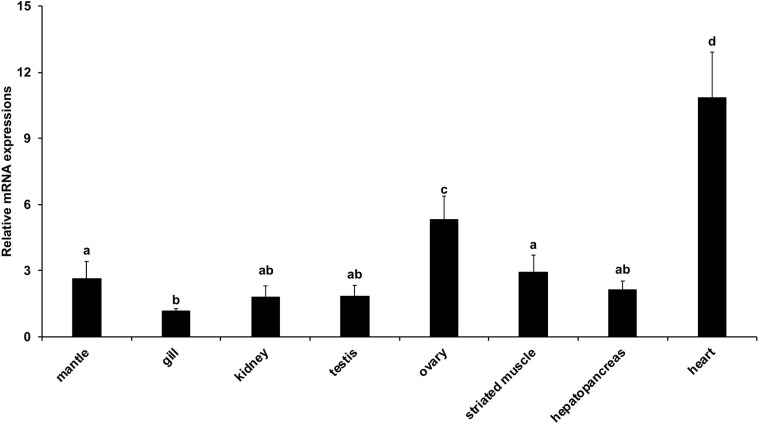
Relative expression levels of *AiPC4* in tissues of bay scallop. Three replicates were performed for each adult tissue and three technical replicates were conducted for each PCR. The comparison of the expression levels of *AiPC4* among different tissues as performed using one way ANOVA followed by with a *post hoc* test. Bars with different superscripts indicate significant differences (*P* < 0.05).

We further investigated the expression patterns of *AiPC4* in the tissues of adult bay scallops (mantle, gill, kidney, testis, ovary, striated muscle, hepatopancreas, and heart) using qRT-PCR data generated from 18 individuals. As shown in Figure 5, *AiPC4* gene was ubiquitously expressed in all the examined tissues; however, the level of expression was predominantly detected in the mantle, ovary, striated muscle, and heart. The expression of *AiPC4* was highest in the heart (9.36-fold compared with the expression in gill), followed by the ovary (4.59-fold), striated muscle (2.55-fold), mantle (2.27-fold), hepatopancreas (1.86-fold), testis (1.61-fold), kidney (1.57-fold), and gill (1.00-fold). The expression levels of *AiPC4* was significantly (*P* < 0.05) higher in the heart and ovary than in the other tissues.

**FIGURE 6:**
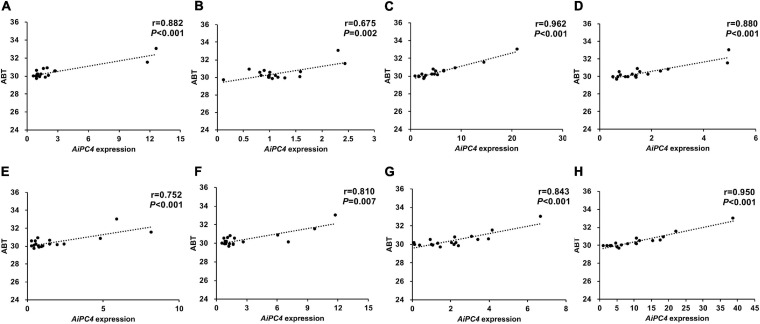
Correlation analysis between *AiPC4* expression levels in tissues and ABT values of bay scallops [**(A)** mantle; **(B)** gill; **(C)** testis; **(D)** ovary; **(E)** kidney; **(F)** striated muscle; **(G)** hepatopancreas; **(H)** heart].

To evaluate the possible role of *AiPC4* in thermal tolerance, we determined the correlation between the ABT values of 18 bay scallops and the *AiPC4* expression levels in their tissues. As shown in Figure 6, the ABT values were significantly (*P* < 0.05) positively correlated with *AiPC4* expression levels in all the tissues examined, with Pearson’s correlation coefficients ranging from 0.675 to 0.962. Moreover, relatively higher correlation coefficients (*r* > 0.850) were observed in the gonads (ovary, 0.880; testis, 0.962) and in temperature sensitive tissues (mantle, 0.882; heart, 0.950). The correlation results indicated that *AiPC4* might be responsible for the regulation of thermal tolerance in bay scallops.

## Discussion

Genome-wide association study has been proven to be a useful approach in elucidating the genetic variation associated with complex quantitative traits and in identifying the genes regulating these traits, thus, providing markers or genes for selective breeding projects (Huang et al., 2010; Korte and Farlow, 2013). In the present study, we employed GLM (Dobson and Barnett, 2012; Hastie, 2017) in PLINK software to evaluate the correlation between SNPs and the ABT of the experimental bay scallops. Previous researches demonstrated that, a quantitative trait affected by multiple genetic loci and non-genetic factors, may be controlled both by major variants with large genetic effects and by minor variants with small effects (Frankham, 1996; Li et al., 2017). Corresponding to this common assumption, the Manhattan plot (Figure 1) in the present study assigned the top 14 SNPs significantly associated with thermal tolerance of bay scallop into several genes at the genome-wide level, indicating that these genes may dominantly contribute together to the thermal tolerance trait with large effects. Some candidate gene families that are closely associated with thermal tolerance in scallops have also been identified by homology alignment in other studies, such as *GRP94* (94 kDa glucose-regulated protein) (Wang et al., 2018a), *TNFR* (tumor necrosis factor receptors) (Xing et al., 2016b), *HSP70* (heat shock protein 70) (Yang et al., 2014), and *metallothionein 1* (Yang et al., 2013). Specifically, four and six SNPs in the promoter region of *HSP70* and *HSP90* were identified to be associated with heat tolerance of two bay scallop populations, *via* elevating transcriptional activities of target genes (Yang et al., 2014, 2015). Similar result in Zhikong scallop has also concluded that the polymorphism at locus + 94 of *CfHSP22* was associated with heat tolerance of scallop (Yang et al., 2011). Above studies including the present research revealed that high expressions of these candidate genes were positively correlated with thermal tolerance, and the mutation at specific loci of the candidate genes (e.g., the SNPs in the promoter region or SNPs in the CDS) might also be associated with thermal tolerance in scallops. In the present study, we conducted a GWAS analysis to screen the possible loci associated with thermal tolerance at the whole genome-wide scale, rather than applied a homology alignment or PCR-based method to detect one or several candidate genes in other studies. In addition, previous studies reported that most of the SNPs in the promoter region affected the putative transcription factor binding sites and this might be the reason of the emergence of different expression patterns of candidate genes under heat stress. However, the molecular mechanism behind thermal tolerance in scallops remains unclear and needs to be further investigated. Therefore, it can be inferred that thermal tolerance in aquatic bivalves is a complex quantitative trait that is regulated by multiple major genes through distinct molecular mechanisms. Among the top 14 significant SNPs identified in the present study, chr15-5091-20379557-1 had the highest correlation with thermal tolerance in bay scallop and was located in *AiPC4*. *AiPC4* has been shown to be involved in RNA polymerase II transcription, repairing DNA damages and stabilizing genome functions (Wang et al., 2004; Mortusewicz et al., 2016). The GWAS result provided an SNP marker that could potentially be used as a thermal tolerance marker in selective breeding for thermal tolerance improvement in bay scallop.

The results of the GWAS showed that thermal tolerance in bay scallop was not dominantly controlled by only a few major genes/loci, but was also regulated by plenty of minor genes/loci, which agreed with the common assumption that quantitative traits are controlled by a small number of genes with large effects and a large number of genes with small effects (Lu et al., 2013). At a less stringent significance level (*P* < 0.05), we obtained 1,906 SNPs in 950 unique genes associated with ABT. These genes were used for GO function annotation and KEGG pathway enrichment analyses. GO and KEGG analyses have been widely utilized in omics data to predict potential gene phenotypes (Zhang et al., 2013) and elucidate important molecular mechanisms (Wang et al., 2019; Feng et al., 2020). In the present study, GO function annotation showed that these genes were primarily annotated in cellular processes, membranes, and binding functions, which were similar to the findings of previous studies on thermal tolerance in other species (Caldwell et al., 2016; Mondal et al., 2019). A reduction in the upper thermal limits of Nile tilapia (*Oreochromis niloticus*) resulted in abnormalities in the cellular processes of erythrocytes (Islam et al., 2020). The reorganization of membrane glycerophospholipids was reported to be crucial for maintaining membrane function in fruit flies (*Drosophila melanogaster*) exposed to thermal stress (Overgaard et al., 2008). Additionally, molecular binding activity is sensitive to temperature alteration (Alison et al., 1996; Schmidt et al., 2000). Furthermore, KEGG pathway enrichment analysis identified the processes influenced by thermal stress. Thermal tolerance research on the land snail, *Theba pisana*, revealed that heat shock protein 90 was more likely to be implicated in signal transduction processes that are activated by heat stress (Mizrahi et al., 2015). Additionally, the metabolic rate of organisms represents a typical response to progressive warming (Abele et al., 2001), and pathogen invasion can have an important effect on their host’s thermal tolerance (Bruneaux et al., 2017). We speculated that, under thermal conditions, bay scallops adapt by altering cellular processes, membrane and binding functions in accordance with the thermal levels, resulting in changes in molecular functions, including binding activity, signal transduction processes, and metabolic rate.

Among the top 14 SNPs identified in this study, chr15-5091-20379557-1, which had the highest correlation with thermal tolerance, was mapped to *AiPC4*. Subsequent investigation of *AiPC4* mutants was conducted by comparing the ABT scallops from the two extreme ends (*N* = 15, 10%) in a cultured population (*N* > 500). Although polymorphism of the chr15-5091-20379557-1 was not verified, multiple sequence alignment analysis identified two additional significant thermal tolerance-associated SNPs (c.234T < C and c.369A < G) located in CDS 1 of *AiPC4*, which was close to the location of chr15-5091-20379557-1. Furthermore, we examined the expression levels of *AiPC4* in different tissues of bay scallop. High expression levels of the gene were detected in multiple thermal-related tissues (Garofalo et al., 2019; Zhou et al., 2019), thus, indicating the important role of *AiPC4* in the hosts thermal tolerance. Temperature is one of the major environmental factors affecting the physiology of aquatic poikilotherms, such as scallops (Zittier et al., 2015). In the present study, *AiPC4* was detected in most of the observed tissues, with high levels of expression in tissues involved in thermal response, such as the heart, striated muscle, and mantle; additionally, the level of expression was high in the ovary, which is necessary for gametogenesis. Scallops possess a typical bivalve heart composed of two auricles and one ventricle, and the contraction of the heart drives its circulatory system, which is necessary for survival under normal and stressful conditions (Shumway and Parsons, 2011). The mantle is considered to be the first barrier against pathogens (Xing et al., 2016b) and is exposed to extrinsic factors, making it more sensitive to temperature variations (Su et al., 2011). Furthermore, swimming performance is mainly controlled by contractile actions (maximal velocity and maximal power output) of the striated adductor muscle. However, the functions of the striated adductor muscle in the bay scallop are strongly influenced by temperature (Olson and Marsh, 1993). Extrinsic factors, such as high temperature, accelerate the oxygen consumption rate of organisms, which could lead to the formation of reactive oxygen species (ROS) and, consequently, result in oxidative damages (Guerra et al., 2012). As reported, PC4 initiates transcriptional activation during TFIIA–TFIID-promoter complex formation and is important for efficient transcription elongation (Kaiser et al., 1995; Calvo and Manley, 2014). In mammals, PC4 acts as a multifaceted factor in oxidative DNA repair and metabolism maintenance (Mortusewicz et al., 2008; Garavís and Calvo, 2017). In particular, changes in environmental factors such as thermal stress can lead to oxidative stress, resulting in cell injury caused by increased ROS. Similar results have also been reported in sea and estuarine animals (*Diplodus vulgaris*, *D. sargus*, *Dicentrarchus labrax*, *Gobius niger*, and *Liza ramada*) (Madeira et al., 2013), Antarctic bivalve *Yoldia eightsii* (Abele et al., 2001), and sea anemone *Anemonia viridis* (Richier et al., 2006). Therefore, we speculated that the relatively high expression level of *AiPC4* in scallops under high temperature stress is necessary for the repair of DNA damages resulted from oxidative stress and the maintenance of genome stability and metabolic functions. Moreover, we observed relatively (*P* < 0.01) higher correlation between ABT values and the expression levels of *AiPC4* in the testis (*r* = 0.962), heart (*r* = 0.950), mantle (*r* = 0.882), ovary (*r* = 0.880), hepatopancreas (*r* = 0.880) and striated muscle (*r* = 0.810) of scallops.

The above observations indicated that the expression levels of *AiPC4* might be responsible for the regulation of thermal tolerance in bay scallop. Although the molecular mechanism of thermal tolerance in mammals has been extensively studied, the regulatory effect of *PC4* in bivalves is poorly understood, thus, making it an area worthy of further research. The findings of this study indicated that *PC4* was involved in the regulation of thermal tolerance in bay scallops.

In conclusion, through GWAS analysis, we identified the most significant SNP associated with thermal tolerance in the bay scallop. The identified SNP was directly assigned to the coding region of *PC4*, which was reported to be an important regulator of DNA damage repair and genome function stabilization. GO function annotation and KEGG pathway enrichment analyses revealed the possible molecular mechanism of thermal tolerance in bay scallops. Expression pattern examination of the target gene, *AiPC4*, and the correlation analysis of gene expression and ABT values jointly demonstrated the importance of *AiPC4* in thermal tolerance in bay scallops. Taken together, these evidences collectively suggest that *PC4* plays a pivotal role in the thermal tolerance in bay scallops.

## Data Availability Statement

The genetic sequencing data involved in our manuscript has been uploaded and deposited in the GenBank (accession number: PRJNA689862).

## Author Contributions

QX, XHu, and ZB conceived and designed the experiments. WL, PL, and JL collected the samples. QX, XZ, JZ, and XHo performed the experiments. XZ, PL, JZ, QZ, and XHo analyzed the data. QX and XZ wrote the manuscript. QX, XZ, and PL revised the manuscript. All authors have read and approved the final manuscript.

## Conflict of Interest

The authors declare that the research was conducted in the absence of any commercial or financial relationships that could be construed as a potential conflict of interest.

## Footnotes

1 http://www.omicshare.com/tools

2 http://www.ebi.ac.uk/Tools/msa/clustalw2/
