# Supplementary material for: Genome-Wide Association Study Reveals PC4 as the Candidate Gene for Thermal Tolerance in Bay Scallop (Argopecten irradians irradians)
Source: Front Genet. 2021 Jul 19;12:650045. doi: 10.3389/fgene.2021.650045 (PMC8328476; doi:10.3389/fgene.2021.650045)
Supplement: Supplementary file 1 [file Data_Sheet_1.PDF]

The detailed procedure on artificial selection of “Haiyifeng 11” from 2011 (G0) to 2016 (G5).

In November 2011, a cultured population with black-brown shell color (N = 50,000) from Laizhou, Yantai (37°10′57 N, 119°56′55 E, Shandong Province, China) and another cultured population with purple-red shell color (N = 50,000) from Jiaonan, Qingdao (35°52′20 N, 120°2′47 E, Shandong Province, China) was collected. Based on shell height of each population, the founder population (G0) of 1,000 bay scallops consisting of 500 (the top 1% in shell height) black-brown shell color individuals from Laizhou, Yantai and another 500 (the top 1% in shell height) purple-red shell color individuals from Jiaonan, Qingdao was constructed. Gonad maturing (Lv et al., 2008) for the 1,000 individuals was conducted from December 2011 to February 2012.

Then artificial spawn and random fertilization of the 1,000 scallops were conducted in March 2012 (G1). After eight months of artificial culture, the top 3% individuals of 100-thousand scallops with purple-red shell color were selected by mass selection based on their shell height in November 2012. Subsequently, a non-lethal method (Mao et al., 2013) was utilized for DNA sampling, 2b-RAD (Wang et al., 2012) sequencing was conducted to identify genome-wide SNPs, and RADtyping software (Fu et al., 2014) was used to perform SNPs calling. After obtaining a high-quality set of SNPs, genetic parameters were evaluated as described in Wang et al (2018) and the estimated breeding value (EBV) of shell height was calculated using our homemade website: http://demo.bbt8.cn:7001/login. In descending order of shell height EBV, the top 10% individuals (N = 300) with inbreeding coefficient less than 0.125 were further selected for next generation reproduction.

In March 2013, artificial spawn and random fertilization of the 300 selected scallops were conducted (G2). After eight months of artificial culture, the procedure of genomic selection (GS) on selection criterion and analysis methods were followed as above mentioned. Then selective breeding of “Haiyifeng 11” was further conducted from 2013 (G2) to 2016 (G5), during which shell height was measured to assess their growth performance, ABT (Xing et al., 2016) was detected to evaluate their thermal tolerance, and the purple-red shell color was regarded as a marked phenotypic trait for the new strain. Simultaneously, survival rate of the new strain in each generation was also calculated in its harvest season. Consequently, the new strain was bred via consecutive generations by selective breeding focusing on traits of shell height, ABT values and shell color from 2011 to 2016.

Reference:

Fu, X., Dou, J., Mao, J., Su, H., Jiao, W., Zhang, L., et al. (2013). RADtyping: An integrated package for accurate *de novo* codominant and dominant RAD genotyping in mapping populations. PLoS One. 8, e79960.

Lv, H., Li, X., Dong, Y., Zhang, G. (2008). The relation between effective accumulated temperature and gonad development period of scallop, *Argopecten irradians*. Mar. Sci. 32(4), 57–60.

Mao, J., Lv, J., Miao, Y., Sun, C., Hu, L., Zhang, R., et al. (2013). Development of a rapid and efficient method for non-lethal DNA sampling and genotyping in scallops. PLoS One. 8, e68096.

Wang, S., Meyer, E., Mckay, J.K., Matz, M. V. (2012). 2b-RAD: A simple and flexible method for genome-wide genotyping. Nat. Methods. 9, 808–810.

Wang, Y., Sun, G., Zeng, Q., Chen, Z., Hu, X., Li, H., et al. (2018b). Predicting growth traits with genomic selection methods in Zhikong scallop (*Chlamys farreri*). Mar. Biotechnol. 20, 769–779.

Xing, Q., Li, Y., Guo, H., Yu, Q., Huang, X., Wang, S., et al. (2016). Cardiac performance: a thermal tolerance indicator in scallops. Mar. Biol. 163, 244.
