# Supplementary figures and images for: Genome-Wide Association Study Reveals PC4 as the Candidate Gene for Thermal Tolerance in Bay Scallop (Argopecten irradians irradians)
Source: Front Genet. 2021 Jul 19;12:650045. doi: 10.3389/fgene.2021.650045 (PMC8328476; doi:10.3389/fgene.2021.650045)

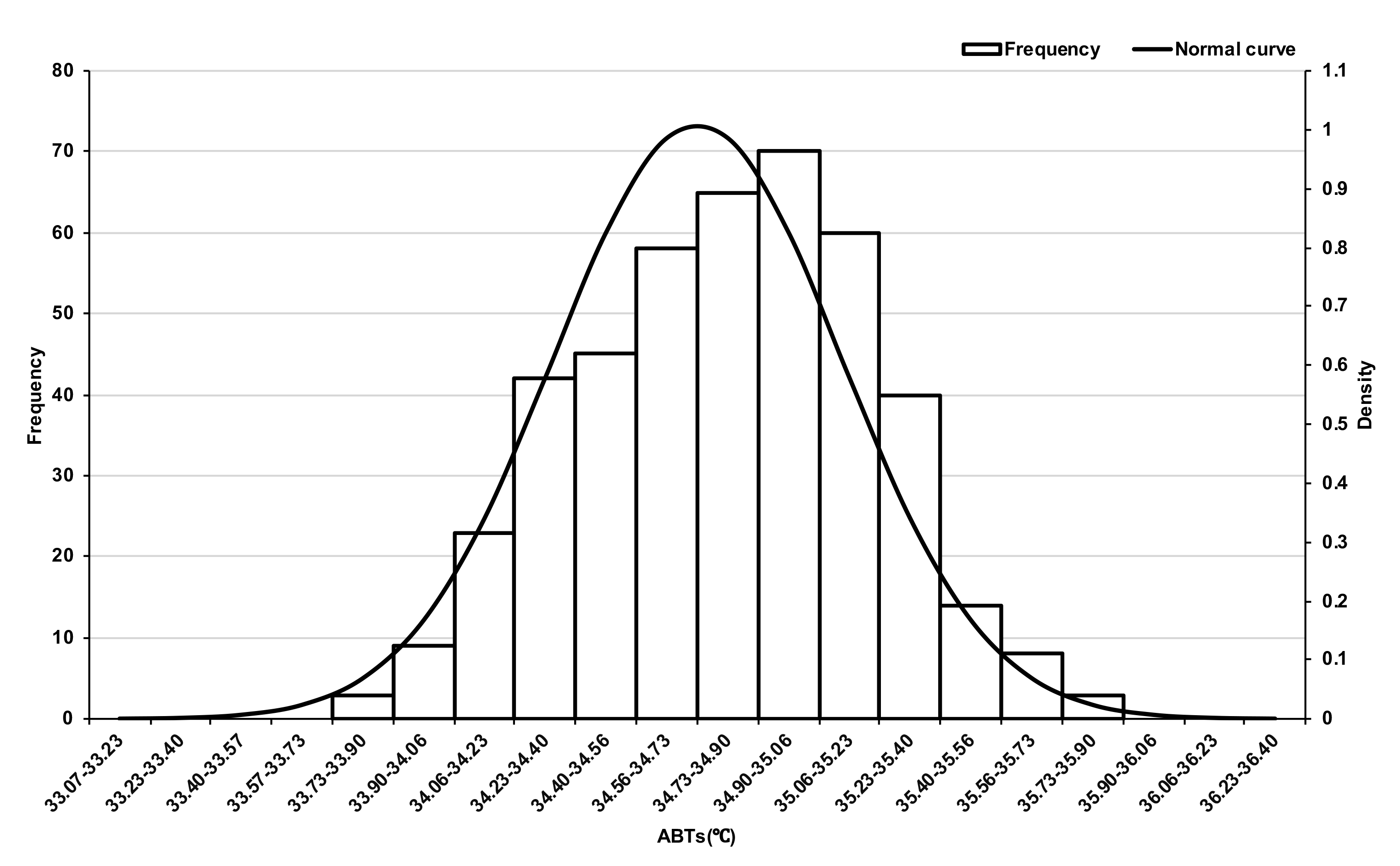

Supplement: Supplementary file 2 [file Image_1.TIF]
